# An engineered non-oxidative glycolytic bypass based on Calvin-cycle enzymes enables anaerobic co-fermentation of glucose and sorbitol by *Saccharomyces cerevisiae*

**DOI:** 10.1186/s13068-022-02200-3

**Published:** 2022-10-17

**Authors:** Aafke C. A. van Aalst, Robert Mans, Jack T. Pronk

**Affiliations:** Department of Biotechnology, Delft University of Technology, Van der Maasweg 9, 2629 HZ Delft, Netherlands

**Keywords:** Ethanol, NADH, Redox engineering, Sorbitol, Yeast, CO_2_, Fermentation, Electrons

## Abstract

**Background:**

*Saccharomyces cerevisiae* is intensively used for industrial ethanol production. Its native fermentation pathway enables a maximum product yield of 2 mol of ethanol per mole of glucose. Based on conservation laws, supply of additional electrons could support even higher ethanol yields. However, this option is disallowed by the configuration of the native yeast metabolic network. To explore metabolic engineering strategies for eliminating this constraint, we studied alcoholic fermentation of sorbitol. Sorbitol cannot be fermented anaerobically by *S. cerevisiae* because its oxidation to pyruvate via glycolysis yields one more NADH than conversion of glucose. To enable re-oxidation of this additional NADH by alcoholic fermentation, sorbitol metabolism was studied in *S. cerevisiae* strains that functionally express heterologous genes for ribulose-1,5-bisphosphate carboxylase (RuBisCO) and phosphoribulokinase (PRK). Together with the yeast non-oxidative pentose-phosphate pathway, these Calvin-cycle enzymes enable a bypass of the oxidative reaction in yeast glycolysis.

**Results:**

Consistent with earlier reports, overproduction of the native sorbitol transporter Hxt15 and the NAD^+^-dependent sorbitol dehydrogenase Sor2 enabled aerobic, but not anaerobic growth of *S. cerevisiae* on sorbitol. In anaerobic, slow-growing chemostat cultures on glucose–sorbitol mixtures, functional expression of PRK-RuBisCO pathway genes enabled a 12-fold higher rate of sorbitol co-consumption than observed in a sorbitol-consuming reference strain. Consistent with the high K_m_ for CO_2_ of the bacterial RuBisCO that was introduced in the engineered yeast strains, sorbitol consumption and increased ethanol formation depended on enrichment of the inlet gas with CO_2_. Prolonged chemostat cultivation on glucose–sorbitol mixtures led to loss of sorbitol co-fermentation. Whole-genome resequencing after prolonged cultivation suggested a trade-off between glucose-utilization and efficient fermentation of sorbitol via the PRK-RuBisCO pathway.

**Conclusions:**

Combination of the native sorbitol assimilation pathway of *S. cerevisiae* and an engineered PRK-RuBisCO pathway enabled RuBisCO-dependent, anaerobic co-fermentation of sorbitol and glucose. This study demonstrates the potential for increasing the flexibility of redox-cofactor metabolism in anaerobic *S. cerevisiae* cultures and, thereby, to extend substrate range and improve product yields in anaerobic yeast-based processes by enabling entry of additional electrons.

**Supplementary Information:**

The online version contains supplementary material available at 10.1186/s13068-022-02200-3.

## Background

With an estimated global output of 103 billion litres in 2021 [1], fuel ethanol produced from plant carbohydrates with the yeast *Saccharomyces cerevisiae* remains the largest process in microbial biotechnology based on product volume. Yeast-based ethanol production is predominantly performed in the USA and Brazil, using corn starch and cane sugar, respectively, as feedstocks [1, 2]. Since the carbohydrate feedstock can contribute up to 70% to the overall process costs of industrial ethanol production, optimization of the ethanol yield on carbohydrates is of paramount importance for process economics [3, 4].

Hydrolysis of corn starch yields glucose as fermentable sugar, while sucrose, the predominant sugar in sugar cane, is hydrolysed to glucose and fructose by yeast invertase [5, 6]. In *S. cerevisiae*, conversion of these hexoses to ethanol and carbon dioxide occurs via the Embden–Meyerhof glycolysis and the fermentation enzymes pyruvate decarboxylase and alcohol dehydrogenase. By producing two moles of ethanol per mole of hexose, this pathway conserves the entire degree of reduction of the substrate in ethanol and, thereby, reaches the theoretical maximum yield of ethanol on hexose sugars [7]. In practice, this theoretical maximum is approached at near-zero growth rates in anaerobic retentostat cultures, in which the impact of yeast biomass formation on carbon and redox metabolism is negligible [8].

To achieve ethanol yields above 2 mol per mole hexose, additional electrons would have to be fed into alcoholic fermentation, for example in the form of NADH. However, the configuration of the metabolic network of wild-type *S. cerevisiae* precludes this option. This constraint is illustrated by experiments in which formate was co-fed to anaerobic, glucose-limited cultures of *S. cerevisiae* strains overproducing the native NAD^+^-dependent formate dehydrogenase Fdh1. In these cultures, the additional electrons provided by formate were channelled into glycerol production rather than into alcoholic fermentation [9]. In *S. cerevisiae*, glycerol production occurs by NADH-dependent reduction of the glycolytic intermediate dihydroxyacetone phosphate to glycerol-3-phosphate by NAD^+^-dependent glycerol-3-phosphate dehydrogenase (Gpd1 or Gpd2). This redox reaction is followed by dephosphorylation of glycerol-3-phosphate by glycerol-3-phosphatase (Gpp1 or Gpp2) [10, 11]. In anaerobic cultures of wild-type *S. cerevisiae* strains, glycerol formation is essential for re-oxidation of ‘surplus’ NADH generated in biosynthetic reactions and has an economically significant negative impact on ethanol yields in industrial processes [12].

The rigidity of the *S. cerevisiae* metabolic network that prevents use of formate-derived NADH for alcoholic fermentation and necessitates glycerol production anaerobic for redox balancing, also prevents anaerobic fermentation of polyols such as mannitol and sorbitol. Mannitol is a main component of brown seaweed, which is investigated as a potential feedstock for ethanol production [13]. Sorbitol occurs in flowering plants [14] and is industrially produced by catalytic hydrogenation of glucose [15]. Although *S. cerevisiae* genomes harbour structural genes for polyol transporters and dehydrogenases, aerobic growth on mannitol and sorbitol typically requires prolonged adaptation [16, 17]. Instantaneous aerobic growth is observed upon combined overexpression of either of the native hexose-transporter genes *HXT13*, *HXT15* or *HXT17* and a native gene encoding mannitol dehydrogenase (*MAN1* or *MAN2*) or sorbitol dehydrogenase (*SOR1* or *SOR2*) [18, 19]. Since these polyol dehydrogenases are NAD^+^-dependent, conversion of mannitol or sorbitol to pyruvate yields one more NADH than glucose upon conversion to pyruvate via the glycolytic pathway. Use of polyols as (co-)substrates therefore provides an interesting model to explore metabolic engineering strategies for feeding additional electrons into yeast-based ethanol production. Such additional electrons could alternatively be provided by, for example, co-feeding of electrochemically produced formate [20, 21] or cathode-associated electrobiotechnology [22, 23].

Our group explored expression of heterologous genes encoding the Calvin-cycle enzymes ribulose-5-phosphate kinase (PRK) and ribulose-1,5-bisphosphate carboxylase/oxygenase (RuBisCO) in yeast to re-route re-oxidation of ‘surplus’ NADH from glycerol formation to ethanol formation [24]. This metabolic engineering strategy encompasses a bypass of the NADH-yielding glyceraldehyde-3-phosphate-dehydrogenase reaction in glycolysis, involving the native non-oxidative pentose-phosphate pathway, PRK and RuBisCO. This bypass allows for redox-neutral synthesis of 3-phosphoglycerate from glucose and CO_2_. Subsequent conversion of 3-phosphoglycerate to ethanol via the regular yeast pathway for alcoholic fermentation then enables re-oxidation of NADH. Implementation of this strategy in engineered strains led to strongly reduced glycerol yields and correspondingly increased ethanol yields on sugar [24]. Strains were further optimized by combined overexpression of non-oxidative pentose-phosphate pathway enzymes [25] to increase supply of ribulose-5-phosphate and deleting the structural gene encoding for the Gpd2 isoenzyme of glycerol-3-phosphate dehydrogenase. This approach yielded *S. cerevisiae* strains with an over 10% higher ethanol yield on glucose in anaerobic batch cultures, while showing the same rates of growth and ethanol production as a non-engineered parental strain [26].

The goal of the present study was to explore whether introduction of a functional PRK-RuBisCO bypass can accommodate the NADH generated upon the entry of sorbitol into glycolysis and, thereby, enable anaerobic (co −)fermentation of this polyol. To this end, Cas9-mediated genome editing was used to construct *S. cerevisiae* strains containing overexpression cassettes for *HXT15* and *SOR2* with or without a simultaneously introduced PRK-RuBisCO bypass. Anaerobic growth and product formation of the resulting engineered strains were quantitatively analysed in anaerobic mixed-substrate batch and chemostat cultures on glucose and sorbitol.

## Results

### Theoretical analysis of glucose or sorbitol fermentation by wild-type and engineered S. cerevisiae

**Fig. 1:**
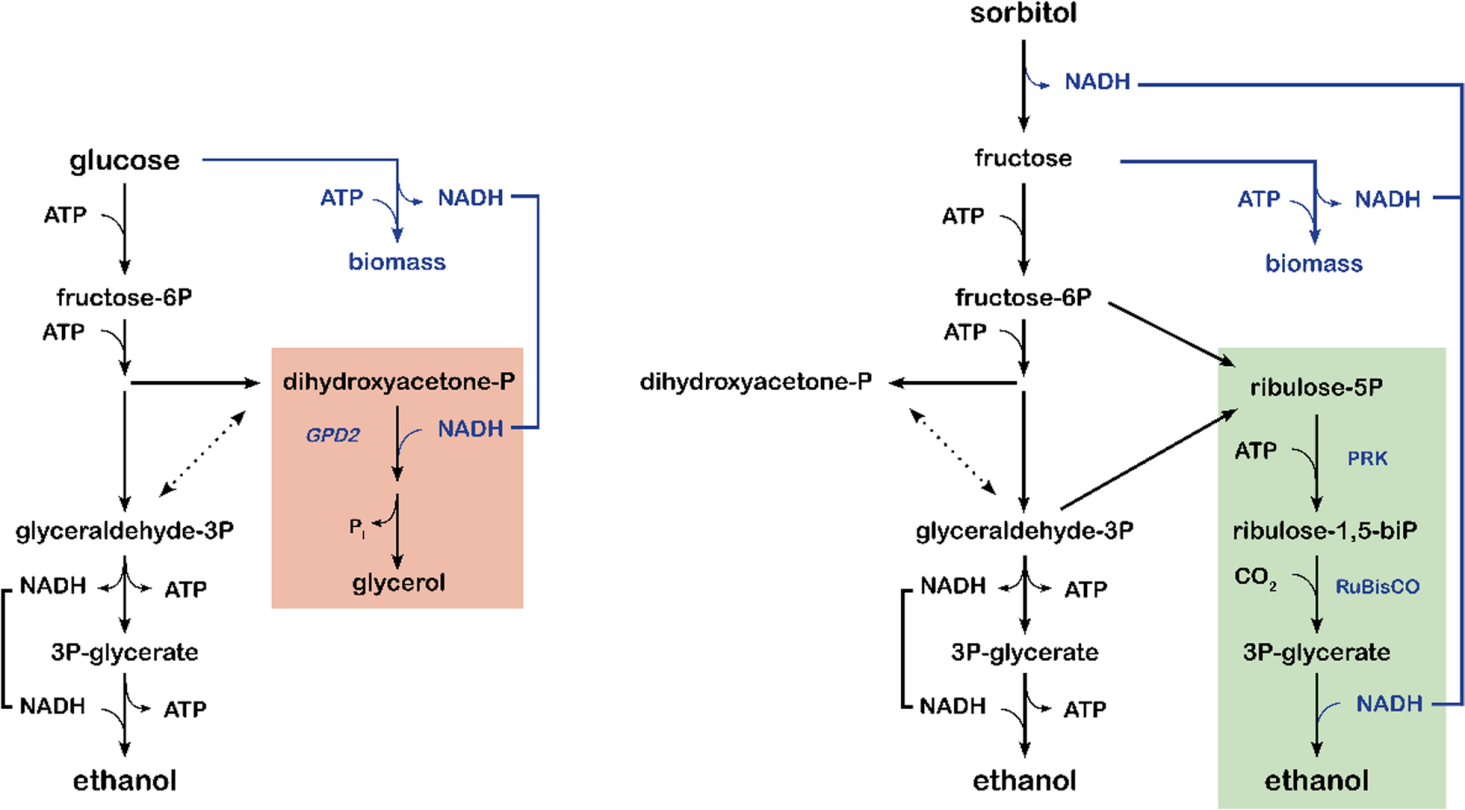
Schematic representation of NADH redox-cofactor balances during glucose fermentation by wild-type *S. cerevisiae* (left) and during sorbitol fermentation by an engineered PRK-RuBisCO expressing strain (right). Coloured boxes indicate pathways used for anaerobic reoxidation of NADH generated in biosynthesis. The red box indicates the native *S. cerevisiae* glycerol pathway for NADH reoxidation, while the green box indicates the engineered PRK-RuBisCO bypass. in the last scenario, re-oxidation of ‘surplus’ NADH generated during biomass formation and/or sorbitol fermentation is coupled to ethanol production. This scenario involves an engineered strain expressing a membrane transporter that enables energy-independent uptake of sorbitol, together with an NAD^+^-dependent sorbitol dehydrogenase and an optimized PRK-RuBisCO pathway. *GPD2*: NAD^+^-dependent glycerol-3-phosphate dehydrogenase; PRK: ribulose-5-phosphate kinase; RuBisCO: ribulose-1,5-bisphosphate carboxylase/oxygenase

Introduction of a PRK-RuBisCO-based ‘bypass’ of the oxidative reaction in glycolysis, as previously applied for improving ethanol yields of anaerobic glucose-grown cultures [24, 26], could theoretically enable redox-neutral fermentation of sorbitol (Fig. 1). Simultaneous operation of the native yeast glycolytic pathway and this bypass should then be redox-cofactor balanced according to the following ‘redox half reactions’:

$$ {3}.{\text{5 sorbitol}} \to {\text{7 ethanol + 7 CO2 + 3}}{\text{.5 NADH + 7 ATP}}\,{\text{via native yeast glycolysis}} $$

$$ {2}{\text{.5 sorbitol + 3}}{\text{.5 NADH}} \to \,{\text{6 ethanol + 3 CO2}}\,\,{\text{via PRK - RuBisCO bypass}} $$

The combined reaction would then provide a redox-balanced, net ATP-generating pathway for anaerobic fermentation of sorbitol (Fig. 1) or, by analogy, mannitol:

$$ {\text{6 sorbitol}} \to \,{\text{13 ethanol + 10 CO2 + 7 ATP}}\,\,{\text{combined}} $$

Functional expression of this pathway in a host organism could, in the absence of growth, support a theoretical maximum yield of 13:6 = 2.17 mol of ethanol per mole sorbitol, which is 8.5% higher than the theoretical yield of ethanol on glucose. Compared to alcoholic fermentation of glucose, this pathway for sorbitol fermentation would yield 42% less ATP per mole of substrate. Provided that sufficient rates of alcoholic fermentation can be achieved to maintain industrially relevant productivities, a low ATP yield on sorbitol could be interesting as it should divert carbon substrate from biomass formation to ethanol production [27, 28].

**Fig. 2:**
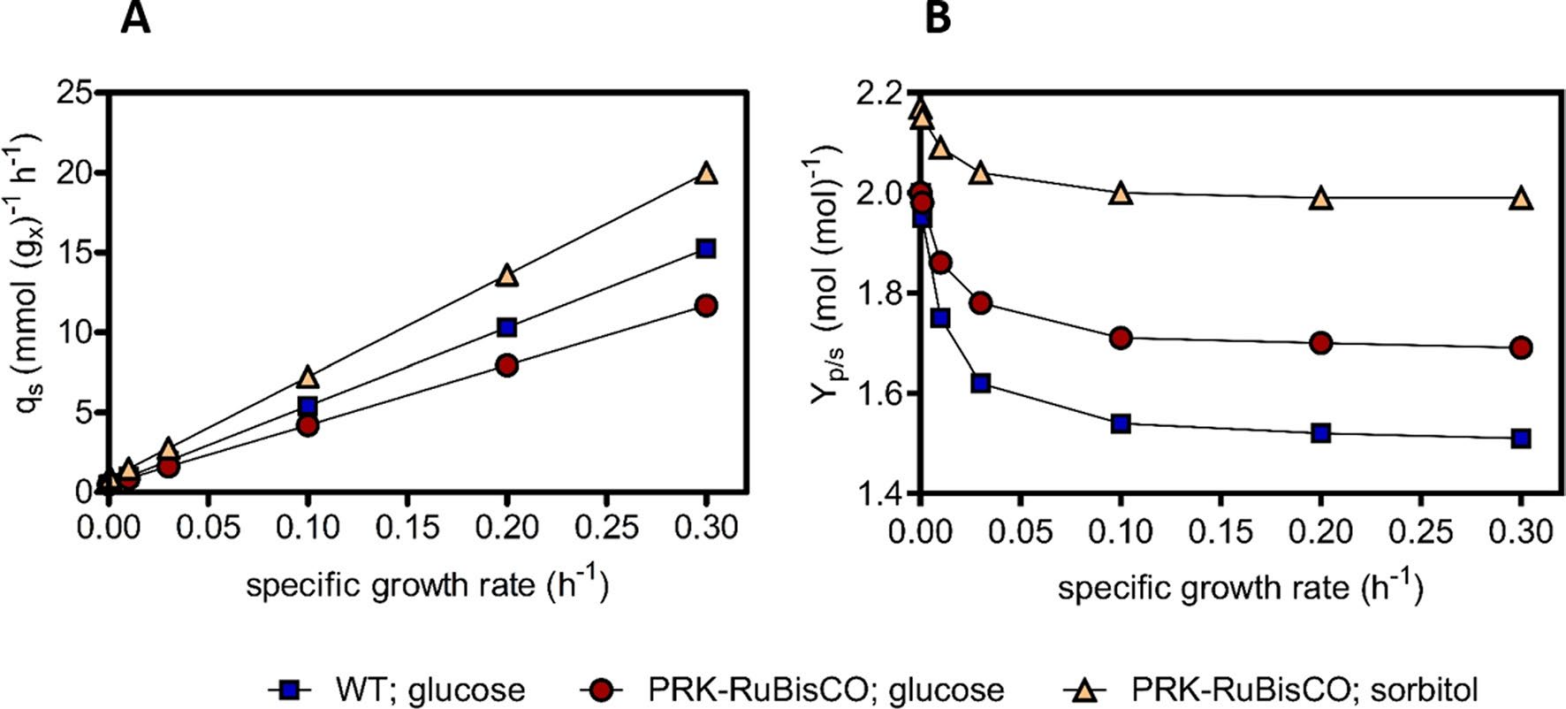
Model-based predictions on kinetics and stoichiometry of anaerobic growth of a reference *S. cerevisiae* strain on glucose and of a strain carrying a functional PRK-RuBisCO-based glycolytic bypass on glucose or on sorbitol as sole carbon source. **A** Biomass-specific substrate-uptake rates and **B** ethanol yield at different growth rates were simulated with an extended stoichiometric model of the core metabolic network of *S. cerevisiae* [27, 29]

To further assess the predicted impact of the proposed metabolic engineering strategy, it was implemented in a stoichiometric model of the core metabolic network of *S. cerevisiae* [27, 29]. The model was then used to calculate biomass and ethanol yields at different specific growth rates. Calculations were based on the assumption that NADH from biomass formation [24, 26], as well as NADH from the reaction catalysed by sorbitol dehydrogenase (Fig. 1), was exclusively re-oxidized by ethanol formation via the PRK-RuBisCO route. Consistent with the calculations presented above, sorbitol fermentation via the engineered pathway was predicted to result in a theoretical maximum yield of 2.17 mol ethanol (mol sorbitol)^−1^ (Fig. 2, Additional file 5: Table S1). Up to a specific growth rate of 0.1 h^−1^, the predicted molar yield of ethanol on sorbitol remained above the theoretical maximum yield on glucose (Fig. 2, Additional file 5: Table S1). As anticipated based on the lower ATP yield from sorbitol fermentation, predicted biomass yields on this substrate (g biomass (mol sorbitol)^−1^) were 42% lower at all specific growth rates than corresponding biomass yields in glucose-grown cultures. At the same specific growth rate, the required biomass-specific rate of sorbitol fermentation was therefore predicted to be 71% higher than in glucose-grown anaerobic cultures.

### Characterization of S. cerevisiae strains overexpressing HXT15 and SOR2

Consistent with results from an earlier study [19], aerobic batch cultures of *S. cerevisiae* IME611, which carried overexpression cassettes for *HXT15* and *SOR2*, grew on synthetic medium (SM) with sorbitol as sole carbon source at a specific growth rate of 0.23 h^−1^. Under the same conditions, cultures of the congenic reference strain IME324 showed virtually no growth (Additional file 5: Table S2). Strain IMX2506, in which overexpression of *HXT15* and *SOR2* was combined with deletion of *GPD2* and introduction of a PRK-RuBisCO pathway optimized for reduced glycerol production in anaerobic glucose-grown batch cultures [26], showed a similar growth rate on sorbitol (0.25 h^−1^). Specific growth rates of these strains on sorbitol were approximately 33% lower than on glucose (0.36 h^−1^ and 0.35 h^−1^, respectively; Table S2). However, despite the fast aerobic growth of strain IMX2506 on sorbitol, no growth was observed after up to 50 days of anaerobic incubation in SM with sorbitol as sole carbon source.

### Co-utilization of glucose and sorbitol in anaerobic batch cultures

**Fig. 3:**
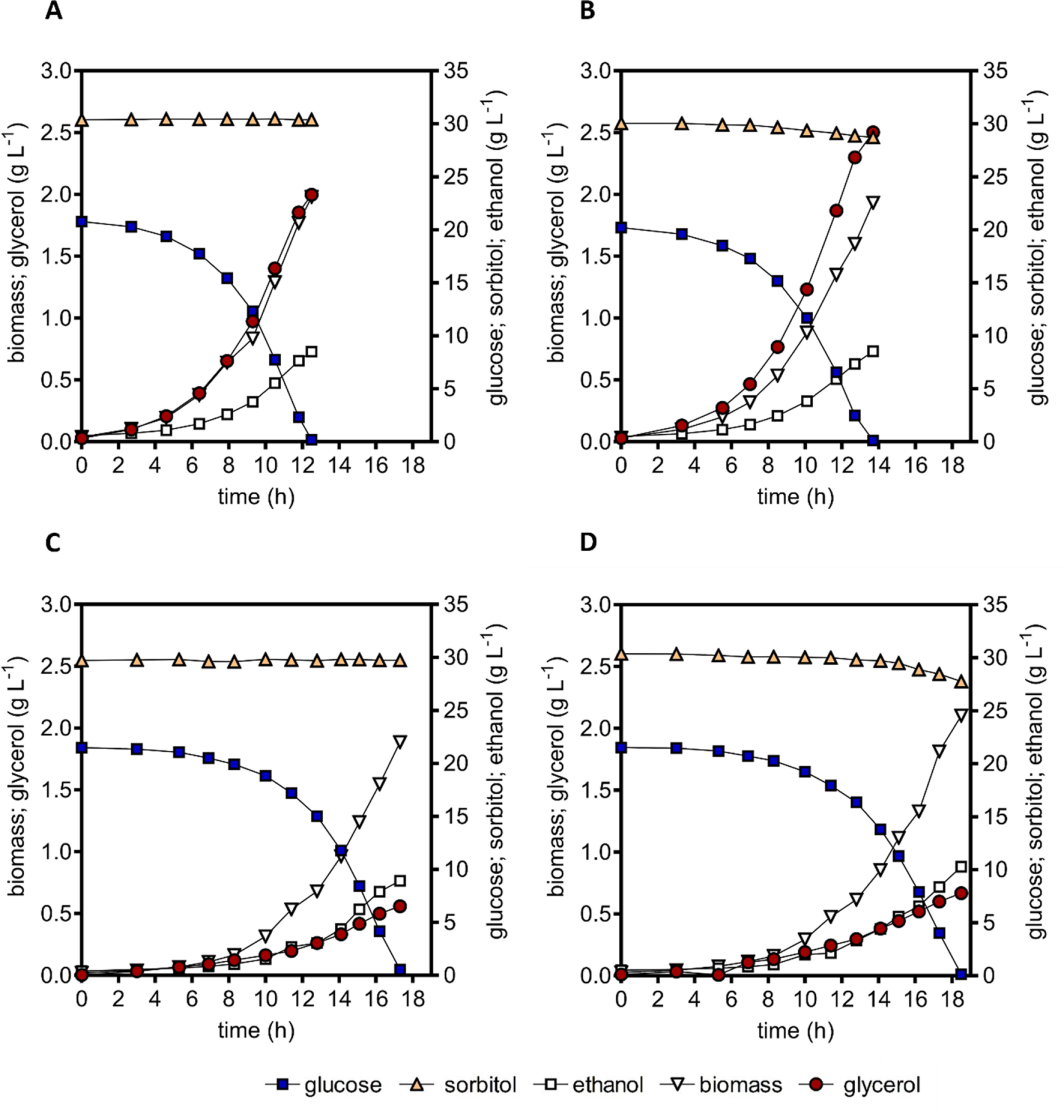
Growth, glucose consumption, sorbitol consumption, ethanol formation and glycerol formation in anaerobic bioreactor batch cultures of *S. cerevisiae* strains IME324 (reference strain) (**A**), IME611 (overexpression cassettes for *HXT15* and *SOR2*) (**B**), IMX1489 (optimized PRK-RuBisCO bypass and *gpd2*∆ mutation, [26]) (**C**) and IMX2506 (optimized PRK-RuBisCO bypass and *gpd2*∆ mutation, overexpression cassettes for *HXT15* and *SOR2*) (**D**). Cultures were grown anaerobically at pH 5 and at 30 °C on synthetic medium containing 20 g L^−1^ glucose and 30 g L^−1^ sorbitol as carbon sources

The inability of *S. cerevisiae* IMX2506 to grow anaerobically on sorbitol as sole carbon source suggested that the in vivo capacity of Hxt15, Sor2 and/or the engineered PRK-RuBisCO bypass was too low to sustain the rate of ATP production required for cellular maintenance. Such a scenario might still allow for anaerobic co-consumption of sorbitol and glucose. We therefore investigated growth in anaerobic bioreactor batch cultures on a mixture of 20 g L^−1^ glucose and 30 g L^−1^ sorbitol (Fig. 3, Table 1). No co-consumption of sorbitol was observed in mixed-substrate cultures of strains IME324 (reference) and IMX1489, which expressed an optimized PRK-RuBisCO bypass and carried a *gpd2∆* mutation (Fig. 3, Table 1). In contrast, upon reaching stationary phase, strain IME611, which carried overexpression cassettes for *HXT15* and *SOR2* had consumed 1.5 g L^−1^ (4.3 mmol g_x_^−1^; subscript x denotes biomass) sorbitol. Co-consumption coincided with a higher glycerol production (16.3 mmoL g_x_^−1^) than observed in strains IME324 and IMX1489 (11.8 mmol g_x_^−1^ and 3.1 mmol g_x_^−1^, respectively, Table 1). This observation indicated that, in strain IME611, the surplus NADH generated during sorbitol co-fermentation (Fig. 1) was predominantly re-oxidized by glycerol formation. In contrast, when strain IMX2506, which combined the genetic modifications carried by strains IME611 and IMX1489, reached stationary phase, consumption of 2.3 g L^−1^ sorbitol (5.4 mmol g_x_^−1^) was accompanied by production of only 3.5 mmol g_x_^−1^ glycerol. In addition, co-consumption of sorbitol by strain IMX2506 coincided with a higher apparent ethanol yield on glucose than observed for the three other strains (Table 1). These results indicated that overexpression of *HXT15* and *SOR2* in a strain with an active PRK-RuBisCO pathway enabled a modest co-fermentation of glucose and sorbitol in anaerobic batch cultures.

**Table 1:** *Yields of biomass and ethanol on glucose, sorbitol consumption, stoichiometric relationships between glycerol production and biomass formation and specific growth rates in anaerobic bioreactor batch cultures of* S. cerevisiae *strains IME324 (reference strain), IME611 (overexpression cassettes for* HXT15 *and* SOR2*), IMX1489 (optimized PRK-RuBisCO bypass and* gpd2*∆ mutation, *[26]*) and IMX2506 (optimized PRK-RuBisCO bypass and* gpd2*∆ mutation, overexpression cassettes for* HXT15 *and* SOR2*)*

Strain	IME324	IME611	IMX1489	IMX2506
*HXT15* and *SOR2* cassettes	no	yes	no	yes
PRK-RuBisCO bypass and *gpd2*∆	no	no	yes	yes
Specific growth rate (^−1^)	0.31 ± 0.01	0.31 ± 0.00	0.30 ± 0.00	0.27 ± 0.00
Biomass yield on glucose (g_x_ mol^−1^)^a^	16.2 ± 0.3	16.0 ± 0.2	16.3 ± 0.9	17.4 ± 0.5
Ethanol yield on glucose (mol mol^−1^)^a^	1.47 ± 0.02	1.51 ± 0.01	1.64 ± 0.04	1.80 ± 0.05
Ethanol yield on substrates (mol mol^−1^)	1.47 ± 0.02	1.42 ± 0.01	1.64 ± 0.04	1.65 ± 0.05
Glycerol produced (mmol (g_x_^−1^))	11.8 ± 0.2	16.3 ± 0.2	3.1 ± 0.1	3.5 ± 0.0
Sorbitol consumed (mmol (g_x_^−1^))	< 0.3	4.3 ± 0.1	< 0.3	5.4 ± 0.2
Degree of reduction recovery (%)	100–102	100–101	100–104	95–99

*Cultures were grown on 20 g L*^*−1*^* glucose and 30 g L*^*−1*^ *sorbitol. ‘Substrates’ refers to the combination of glucose and sorbitol. Specific growth rates and stoichiometries were calculated from at least 7 sampling points in the exponential growth phase. Values represent averages* ± *mean deviations of measurements on independent duplicate cultures for each strain. Degree of reduction balances yielded electron recoveries between 95 and 104%*

^a^These yield values on glucose also include ethanol and biomass formed by the additional consumption of sorbitol

### Co-fermentation of sorbitol by anaerobic mixed-substrate chemostat cultures

**Fig. 4:**
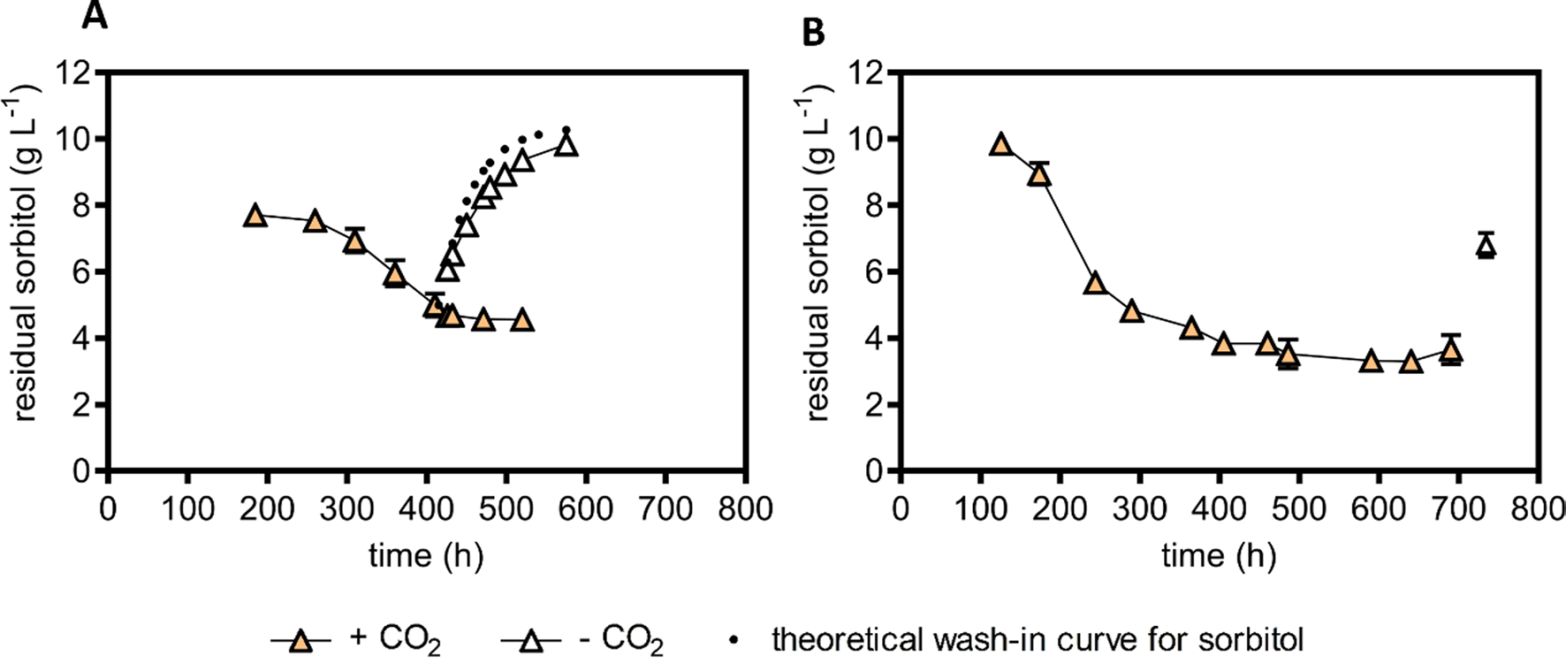
Sorbitol consumption by anaerobic chemostat cultures of *S. cerevisiae* strains IMX2506 (optimized PRK-RuBisCO bypass and *gpd2*∆ mutation, overexpression cassettes for *HXT15* and *SOR2*) (**A**) and IMX1489 (optimized PRK-RuBisCO bypass and *gpd2*∆ mutation [26]) (**B**). Chemostat cultures were grown at a dilution rate of 0.025 h^−1^ on 10 g L^−1^ of glucose and 10 g L^−1^ of sorbitol. **A** Average residual sorbitol concentration ± standard deviation in four chemostat cultures of strain IMX2506. After 400 h, the CO_2_ content of the inlet gas was reduced to zero in two of the four cultures. The dotted line represents expected wash-in kinetics of sorbitol in the absence of sorbitol consumption: c(t) = c_in_-((c_in_-c_400_)*e^(−D*t))^ with c = residual sorbitol concentration, c_400_ = sorbitol concentration at 400 h, $\hbox{c}_{{\rm in}}$ = sorbitol concentration in medium feed and D = dilution rate. **B**: Average sorbitol concentration ± standard deviation in two chemostats of strain IMX1489. CO_2_ supplementation was stopped at 700 h

In anaerobic mixed-substrate batch cultures of strain IMX2506, sorbitol consumption predominantly occurred when the supplied glucose was already nearly consumed (Fig. 3, panel D). Based on this observation, we hypothesized that glucose and sorbitol competed for Hxt transport proteins. Sorbitol co-consumption at low glucose concentrations was investigated in anaerobic chemostat cultures. In chemostat cultures, culture broth is removed at a fixed flow rate (F_out_) while the culture volume (V_L_) is kept constant by continuous supply of fresh medium, thus controlling the specific growth rate [30, 31]. The anaerobic chemostat cultures were grown on a mixture of 10 g L^−1^ glucose and 10 g L^−1^ sorbitol at a dilution rate (F_out_/V_L_ which, in steady-state cultures, equals specific growth rate) of 0.025 h^−1^. In these chemostat cultures, residual glucose concentrations were below 0.036 g L^−1^. In chemostat cultures of strain IMX2506, anaerobic sorbitol conversion rates were 12-fold higher than in cultures of strain IME611, which carried overexpression cassettes for *HXT15* and *SOR2* but did not harbour a PRK-RuBisCO bypass (Table 2). In cultures of the congenic reference strain IME324, which carried no PRK-RuBisCO bypass or *HXT15* and *SOR15* overexpression cassettes (Table 2), glucose was virtually completely consumed but sorbitol concentrations in the chemostat cultures equalled those in the medium inflow. Th99999is observation reflected the reference strain’s inability to anaerobically consume sorbitol. However, the PRK-RuBisCO-expressing reference strain IMX1489, which did not contain overexpression cassettes for *HXT15* and *SOR15*, did co-consume sorbitol (Table 2, Fig. 4B).

**Table 2:** Yields of biomass and ethanol on glucose, sorbitol consumption, biomass-specific sorbitol uptake rates and stoichiometric relationships between glycerol production and biomass formation in anaerobic bioreactor chemostat cultures of *S. cerevisiae* strains IME324 (reference strain), IME611 (overexpression cassettes for *HXT15* and *SOR2*), IMX1489 (optimized PRK-RuBisCO bypass and *gpd2*∆ mutation, [26]) and IMX2506 (optimized PRK-RuBisCO bypass and *gpd2*∆ mutation, overexpression cassettes for *HXT15* and *SOR2*). ‘Substrates’ refers to the combination of glucose and sorbitol. Cultures were grown at a dilution rate of 0.025 h^−1^ on 10 g L^−1^ of glucose and 10 g L^−1^ of sorbitol (pH 5)

Strain	IME324	IME611	IMX1489	IMX2506
*HXT15* and *SOR2* cassettes	no	yes	no	yes
PRK-RuBisCO bypass and *gpd2*∆	no	no	yes	yes
Biomass yield on substrates (g_x_ mol^−1^)	14.2 ± 0.1	14.3 ± 0.7	12.0 ± 0.9	13.3 ± 0.4
Yield of ethanol on glucose (mol mol^−1^)^a^	1.62 ± 0.03	1.66 ± 0.07	2.96 ± 0.00	2.86 ± 0.02
Ethanol yield on substrates (mol mol^−1^)	1.63 ± 0.04	1.61 ± 0.06	1.76 ± 0.01	1.83 ± 0.04
Glycerol/biomass (mmol g_x_^−1^)	8.1 ± 0.2	10.8 ± 0.5	0.6 ± 0.2	1.0 ± 0.0
Sorbitol consumed (mmol g_x_^−1^)	< 0.5	2.2 ± 1.1	33.9 ± 3.0	27.0 ± 0.1
Biomass-specific sorbitol uptake rate (mmol g_x_^−1^ h^−1^)	< 0.02	0.06 ± 0.03	0.89 ± 0.08	0.67 ± 0.00
Degree of reduction recovery (%)	99–100	98–102	98–99	100–101

Values represent averages ± mean deviations of measurements on independent steady-state triplicate cultures of strain IME611 and duplicate cultures of strain IMX2506

^a^These apparent yield values on glucose also include ethanol and biomass formed by the additional consumption of sorbitol

The *Thiobacillus denitrificans* form-II RuBisCO present in strains IMX1489 and IMX2506 has a high K_m_ for CO_2_ (0.26 mM; [32]). To verify involvement of the PRK-RuBisCO pathway in the increased sorbitol consumption by engineered yeast strains, the inlet gas, which routinely consisted of a mixture of 90% N_2_ and 10% CO_2_, was switched to pure N_2_ during fermentation runs. This switch led to an instantaneous, progressive increase of the residual sorbitol concentration, with a profile that closely corresponded to wash-in kinetics in the complete absence of sorbitol consumption (Fig. 4). These results, in combination with the absence of sorbitol consumption in strain IME611 lacking the PRK-RuBisCO pathway (Table 2), confirmed involvement of in vivo RuBisCO activity in anaerobic sorbitol co-fermentation.

### Prolonged continuous cultivation on a glucose–sorbitol mixture does not select for improved sorbitol fermentation

**Fig. 5:**
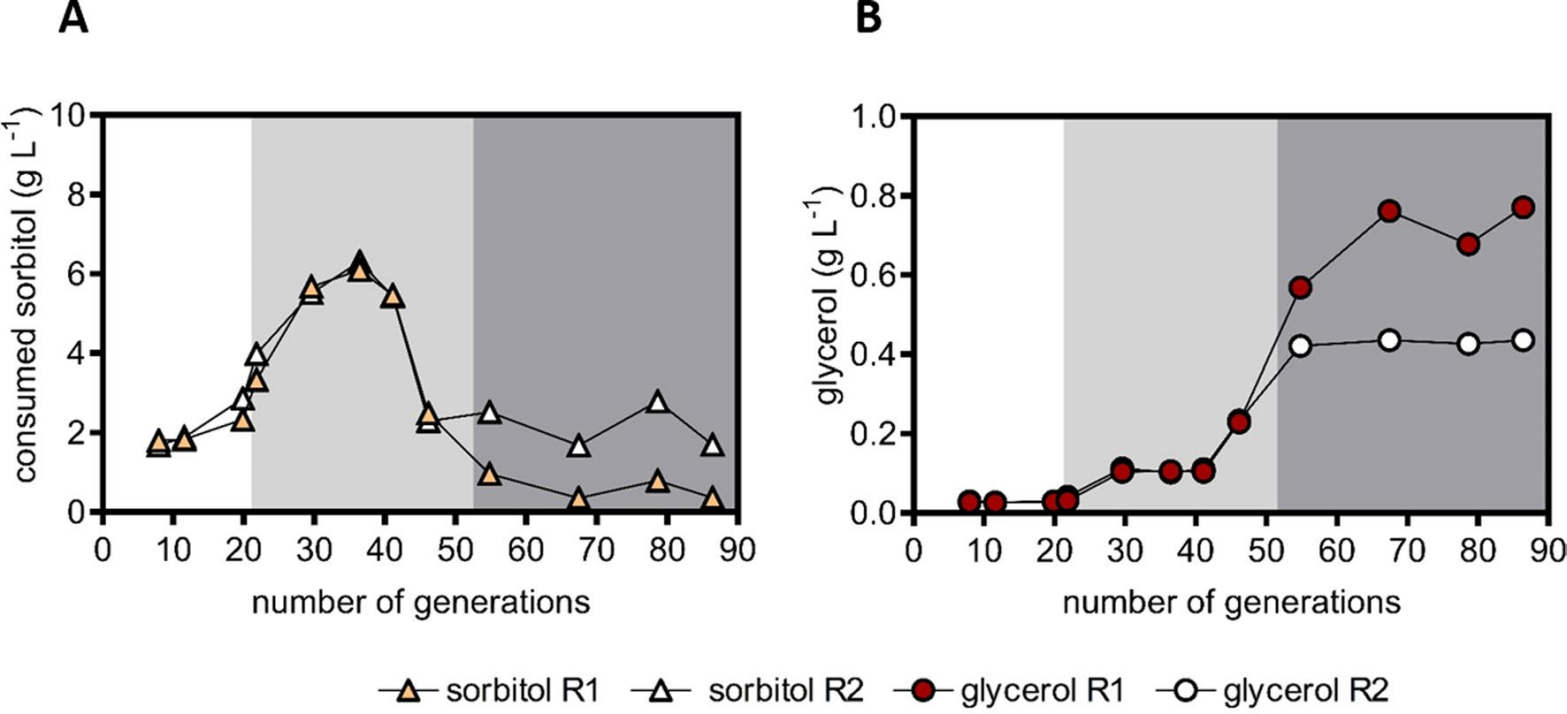
Sorbitol consumption (**A**) and glycerol production (**B**) in duplicate anaerobic chemostat cultures of strain IMX2506 (optimized PRK-RuBisCO bypass and *gpd2*∆ mutation, overexpression cassettes for *HXT15* and *SOR2*). For the first 20 generations, the cultures were grown on a mixture of 10 g L^−1^ sorbitol and 10 g L^−1^ glucose at a dilution rate of 0.05 h^−1^. After 20 h, the dilution rate was decreased to 0.025 h^−1^ and after 50 generations the substrate mixture was changed to 20 g L^−1^ sorbitol and 10 g L^−1^ glucose

During anaerobic chemostat cultivation of strain IMX2506 on sorbitol and glucose, sorbitol co-consumption increased during the first 10 volume changes. After this point, it stabilized for approximately 3 volume changes (Fig. 4A), leaving ~ 4 g L^−1^ of sorbitol unused. Based on the assumption that improved co-consumption of sorbitol by spontaneous mutants would confer a selective advantage, two new anaerobic continuous cultures were grown on a glucose and sorbitol mixture for over 80 generations. During the first approximately 40 generations, sorbitol consumption by the mixed-substrate chemostat cultures improved. However, contrary to expectation, sorbitol co-consumption deteriorated rather than improved further in both evolution experiments. In evolution line 1, sorbitol co-consumption completely ceased, while in evolution line 2 its rate declined by approximately 3.5-fold (Fig. 5A). In both prolonged continuous cultures, glycerol production increased, indicating a decline of the in vivo activity of the PRK-RuBisCO pathway (Fig. 5B).

To explore underlying mechanisms for the reduced sorbitol co-consumption observed during prolonged mixed-substrate cultivation, whole-genome sequencing was performed on culture samples. After 86 generations, sequence data from both chemostat cultures showed an identical non-synonymous point mutation in the open-reading frame of the spinach *prk* sequence, which caused an alanine-to-aspartate change at position 193 in the Prk protein (A193D). In evolution line 1, in which sorbitol co-consumption was virtually completely abolished, 73% of the *prk* sequence reads carried the mutation. A lower percentage (45%) of reads carrying this mutation was identified in evolution line 2, in which some sorbitol co-consumption was still observed after 86 generations. Sequence alignment showed that the mutation in *prk* involves a highly conserved amino acid residue in type II PRK proteins from 49 different species (Archaea, Eukaryotes and Cyanobacteria; position 372 in the multiple sequence alignment as identified in Additional file 5: Fig. S6 of the publication by Gurrieri et al. [33]). In these dimeric type II PRK enzymes (including the spinach PRK expressed in this study), an alanine residue is invariably found at this position, while the bacterial allosterically regulated and octameric type I PRK enzymes, either have an alanine or a threonine in this position. For both PRK types, this residue is located on the edge of a β-sheet [33, 34]. We hypothesize that exchange of a neutral residue (alanine/threonine) for a negatively charged residue (aspartate) in a highly conserved position disrupted protein folding and thereby reduced or even abolished PRK activity.

No sequence changes were found in other coding regions in the analysed culture samples where sorbitol consumption was greatly reduced after prolonged cultivation. However, sequence data from both evolution experiments displayed segmental aneuploidy in multiple chromosomes (Additional file 5: Fig. S1, Table S3). Some amplifications differed between the two experiments or, based on read coverages, only occurred in part of the population. Since the duplicated regions carried multiple genes, no definitive interpretation is possible without extensive reverse engineering studies [35, 36]. However, we note that ca. 100 kb duplication of a fragment of chromosome IV (~ 1,075,000–1,175,000) that was found in both evolution experiments carried the *HXT6* and *HXT7* genes. These genes, which encode the major high-affinity glucose transporters of *S. cerevisiae*, are highly expressed in glucose-limited chemostat cultures [37]. A large duplication of a centromeric region of chromosome XV (~ 0–495,000) carried the *GPD2* locus into which the overexpression cassettes for six genes encoding non-oxidative pentose-phosphate-pathway enzymes had been integrated. In addition, this region harboured *HXT11* which, like *HXT15*, encodes a functional sorbitol transporter [19].

## Discussion

Introduction of functional Calvin-cycle enzymes in *S. cerevisiae* was previously shown to enable couple re-oxidation of ‘surplus’ NADH, generated during biomass formation in anaerobic cultures, to ethanol formation, thereby reducing glycerol production and enhancing ethanol yield on sugars [24, 26, 38, 39]. The present study demonstrates that expression of a non-oxidative, PRK-RuBisCO bypass of glycolysis enabled a 12-fold higher rate of sorbitol conversion in anaerobic chemostat cultures grown on glucose–sorbitol mixtures than observed in cultures of a reference *S. cerevisiae* strain.

As reported for aerobic growth of *S. cerevisiae* on sorbitol [19], overexpression of *HXT15* and *SOR2* was required to achieve a modest co-utilization of sorbitol by anaerobic batch cultures of *S. cerevisiae* grown on glucose–sorbitol mixtures (Fig. 3). In contrast, constitutive overexpression of these genes was not required for a much more extensive sorbitol co-consumption by slow-growing, glucose-limited anaerobic chemostat cultures of PRK-RuBisCO-expressing strains (Fig. 4B). Acquisition of the ability to aerobically consume sorbitol in batch cultures was previously attributed to mutations in the Tup1-Cyc8 complex, that relieve transcriptional repression of many genes, including *HXT15* and *SOR2* [40]. However, whole-genome sequencing did not identify such mutations in steady-state chemostat cultures of the PRK-RuBisCO-expressing reference strain IMX1489, which co-consumed sorbitol but did not contain overexpression cassettes for *HXT15* and *SOR15*. Gene expression patterns in *S. cerevisiae* are strain dependent [41] and glucose concentrations strongly affect transcriptional regulation of *HXT* transporter genes [37]. Furthermore, expression of *HXT13* and *HXT15* (two polyol transporters) has been linked to growth on non-fermentable carbon sources [42]. We hypothesize that the conditions in our chemostat setup, which combined low residual glucose concentrations with the presence of sorbitol, stimulated expression of native *HXT13* and *HXT15* genes and thereby enabled sorbitol utilization in strain IMX1489 (Fig. 4B).

Although expression of the PRK-RubisCO bypass enabled anaerobic co-consumption of sorbitol and glucose, anaerobic growth of *S. cerevisiae* on sorbitol as sole carbon source was not yet achieved. In order for sorbitol fermentation to sustain anaerobic growth, the biomass-specific rate of ATP formation should exceed the cellular ATP requirement of anaerobic cultures for cellular maintenance processes (approximately 1 mmol ATP (g biomass)^−1^ h^−1^; [8]). At an ATP stoichiometry of 1.17 mol ATP (mol sorbitol)^−1^ calculated for the PRK-RuBisCO-dependent pathway, the threshold biomass-specific rate of sorbitol fermentation for anaerobic growth would then equal 0.86 mmol (g biomass)^−1^ h^−1^. The biomass-specific sorbitol uptake rate of strain IMX2506 in anaerobic mixed-substrate chemostat cultures of 0.67 mmol sorbitol g_x_^−1^ h^−1^ (Table 4) remained below this threshold.

In aerobic batch cultures of *HXT15-* and *SOR2*-overexpressing *S. cerevisiae* strains (IME611 and IMX2506) (Additional file 5: Fig. S2) a specific growth rate of 0.22 h^−1^ was observed. At an estimated biomass yield of 0.07 g_x_^−1^ (mmol sorbitol)^−1^ in these respiro-fermentative cultures, this growth rate would correspond to a biomass-specific sorbitol-consumption rate of 3.0 mmol (g biomass)^−1^ h^−1^. In anaerobic cultures, such an in vivo activity of sorbitol transport and oxidation to fructose would be over threefold higher than required to meet maintenance-energy requirements.

Alternatively, in vivo capacity of the PRK-RuBisCO pathway might be too low to support anaerobic growth on sorbitol alone. Since form-II RuBisCO enzymes such as *T. denitrificans* cbbM exhibit low k_cat_ values [32] and require chaperones for functional expression, high-level expression is required to support the in vivo fluxes required for anaerobic growth on sorbitol as sole carbon source. Implementation of RuBisCO variants with a higher k_cat_ [43], potentially in combination with increased expression of PRK, may enable anaerobic growth on sorbitol.

Instead of targeted engineering of the PRK-RuBisCO pathway, we tried to use adaptive laboratory evolution to identify key genes involved in its in vivo capacity. While, in two parallel evolution experiments, the degree of sorbitol co-consumption initially increased, it subsequently deteriorated. This deterioration was accompanied by an increase of the glycerol concentration in the cultures that indicated loss of functionality of the PRK-RuBisCO pathway. No mutations or reduction of copy number of the *cbbM* expression cassettes were observed upon prolonged cultivation. This observation suggested that protein burden caused by RuBisCO overexpression was unlikely to be a key factor in the unexpected loss of pathway functionality. Instead, a mutation in the *prk* gene, duplication of overexpression cassettes for non-oxidative pentose phosphate pathway enzymes and duplication of a genomic region carrying the *HXT6* and *HXT7* genes that both encode the high-affinity glucose transporters, were found*.* These mutations may indicate a trade-off between kinetics of sorbitol fermentation via the PRK-RuBisCO bypass and kinetics of glucose uptake at the very low residual glucose concentrations in the chemostat cultures. Further research is required to investigate whether this trade-off is impacted by the reported toxic effects of PRK overexpression [44].

## Conclusion

This study provides a proof of principle for engineering a redox-cofactor-neutral bypass of glycolysis to enable entry of additional electrons in the main yeast alcoholic fermentation pathway. This strategy enabled a higher theoretical maximum yield of ethanol than possible with sugars as sole source of electrons. In addition to further analysis of the rate-controlling steps in the PRK-RuBisCO pathway, alternative bypasses of the oxidative step in glycolysis with a better ATP stoichiometry may be explored. A particularly interesting option is offered by the combined expression of a heterologous phosphoketolase, phosphotransacetylase and acetylating acetaldehyde dehydrogenase [45, 46]. In contrast to the PRK-RuBisCO strategy, this redox-cofactor-neutral bypass of glyceraldehyde-3-phosphate dehydrogenase has a net positive ATP yield [27]. In addition to improving polyol fermentation, further research on extending flexibility of redox-cofactor balancing in yeast may ultimately enable the co-consumption of auxiliary electron donors such as formic acid and/or hydrogen [9, 47, 48] to boost ethanol yields on sugars beyond current limits.

## Methods

### Strains and maintenance

*Saccharomyces cerevisiae* strains used in this study (Table 3) belong to the CEN.PK lineage [49, 50] and were propagated in YPD medium (10 g L^−1^ Bacto yeast extract, 20 g L^−1^ Bacto peptone, 20 g L^−1^ glucose). *Escherichia coli* XL-I Blue-derived strains were propagated in LB medium (10 g L^−1^ Bacto tryptone, 5 g L^−1^ Bacto yeast extract, 20 g L^−1^ glucose) supplemented with 100 µg mL^−1^ ampicillin. After addition of sterile glycerol (30% v/v) to late exponential-phase *S. cerevisiae* cultures or overnight *E. coli* cultures, 1-mL aliquots were frozen and stored at −80 °C.

**Table 3:** S. cerevisiae *strains used in this study*

Strain name	Relevant genotype	Parental strain	Reference
CEN.PK113-7D	*MATa URA3 GPD2*	-	[49]
IMX581	*MATa ura3-52 GPD2 can1::cas9-natNT2*	CEN.PK113-5D	[51]
IME324	*MATa ura3-52 GPD2 can1::cas9-natNT2* p426-*TEF* (*URA3*)	IMX581	[26]
IMX1489	*MAT2 ura3-52 can1::cas9-natNT2 gpd2::*p*TDH3-RPE1-*t*RPE1*, p*PGK1-TKL1-*t*TKL1*, p*TEF1-TAL1-*t*TAL1*, p*PGI1-NQM1-*t*NQM1*, p*TPI1-RKI1-*t*RKI1*, p*PYK1-TKL2-*t*TKL2 sga1::*p*DAN1-prk,* p*TDH3-cbbm-*t*CYC1* (9 copies), p*TPI1*-*groES-*t*PGI1*, p*TEF1*-*groEL-*t*ACT1* pUDR103 (*KlURA3*)	IMX581	[26]
IMX2411	*MATa ura3-52 GPD2 can1::cas9-natNT2 X-2::*p*TEF1-HXT13-tCYC1,* p*ACT1-SOR2-*t*CPS1*	IMX581	This study
IMX2495	*MAT2 ura3-52 can1::cas9-natNT2 gpd2::*p*TDH3-RPE1-*t*RPE1*, p*PGK1-TKL1-*t*TKL1*, p*TEF1-TAL1-*t*TAL1*, p*PGI1-NQM1-*t*NQM1*, p*TPI1-RKI1-*t*RKI1*, p*PYK1-TKL2-*t*TKL2 sga1::*p*DAN1-prk,* p*TDH3-cbbm-*t*CYC1* (9 copies), p*TPI1*-*groES-*t*PGI1*, p*TEF1*-*groEL-*t*ACT1 X-2::*p*TEF1-HXT13-tCYC1,* p*ACT1-SOR2-*t*CPS1*	IMX1489	This study
IMX2506	*MAT2 ura3-52 can1::cas9-natNT2 gpd2::*p*TDH3-RPE1-*t*RPE1*, p*PGK1-TKL1-*t*TKL1*, p*TEF1-TAL1-*t*TAL1*, p*PGI1-NQM1-*t*NQM1*, p*TPI1-RKI1-*t*RKI1*, p*PYK1-TKL2-*t*TKL2 sga1::*p*DAN1-prk,* p*TDH3-cbbm-*t*CYC1* (9 copies), p*TPI1*-*groES-*t*PGI1*, p*TEF1*-*groEL-*t*ACT1 X-2::*p*TEF1-HXT13-tCYC1,* p*ACT1-SOR2-*t*CPS1* p426-*TEF* (*URA3*)	IMX2495	This study
IME611	*MATa ura3-52 GPD2 can1::cas9-natNT2 X-2::*p*TEF1-HXT13-tCYC1,* p*ACT1-SOR2-*t*CPS1* p426-*TEF* (*URA3*)	IMX2411	This study

### Construction of plasmids and expression cassettes

Cas9-based genome editing [51] was employed for markerless integration of expression cassettes in the intergenic region X-2 [52]. Oligonucleotide primers used in this study are listed in Table S1. Plasmid fragments and yeast integration cassettes were PCR amplified with Phusion High Fidelity DNA polymerase (Thermo Fisher Scientific, Waltham MA) and Dreamtaq polymerase (Thermo Fisher) was used for diagnostic PCR. Yeast genomic DNA was isolated as described by [53]. PCR-amplified DNA fragments were purified from agarose gels with a ZymoClean Gel DNA kit (Zymo Research, Irvine CA) or purified directly from the PCR mix with a GeneJET kit (Thermo Fisher). Purified plasmid backbone and insert fragments were assembled with a Gibson assembly cloning kit (New England Biolabs, Ipswich MA), downscaled to 5-µL reaction volume. After plasmid assembly, 1 µL of the resulting mixture was used for heat-shock transformation of *E. coli* XL-I Blue [54]. Plasmids were isolated from *E. coli* using Sigma GenElute Plasmid kit (Sigma-Aldrich).

Plasmids used and constructed in this study are listed in Table 4. A p*TEF1*-*HXT15*-t*CYC1* expression cassette was obtained by assembling three DNA fragments. A *TEF1* promoter fragment and a *CYC1* terminator fragment were amplified from p426-TEF [55] with primer pairs 16711/17031 and 17032/16712, respectively. The coding region of *HXT15* ORF was amplified with primer pair 16705/16706 from genomic DNA of strain CEN.PK113-7D. Assembly of these three fragments by homologous recombination of terminal sequences introduced during amplification yielded a p*TEF1*-*HXT15*-t*CYC1* expression cassette for integration at the X-2 locus [52].

**Table 4:** Plasmids used and constructed in this study

Name	Characteristics	Origin
pUDR538	*Hyg*, gRNA.*X-2*–2 µm-gRNA.*X-2*	[56]
p426-*TEF*	2 µm, *URA3*, p*TEF1*-t*CYC1* (empty vector)	[55]
pUD968	2 µm, *URA3*	This study
pUDE885	2 µm, *URA3*, p*ACT1-*t*CPS1*	This study
pUDE941	2 µm, *URA3*, p*ACT1*-*SOR2-*t*CPS1*	This study

To remove its p*TEF1* and t*CYC1* promoter and terminator sequences, p426-TEF [55] was used as template for PCR amplification with primer pairs 15514/10901 and 15515/7388. The amplified fragments were purified and digested with KpnI and PfoI (Thermo Fisher). Digestion products were repurified and ligated with T4 DNA ligase (Thermo Fisher), yielding pUD968. p*ACT1* and t*CPS1* sequences were amplified from genomic DNA of strain CEN.PK113-7D with primer pairs 15548/15549 and 15550/15551, respectively. Gibson assembly of the resulting fragments with KpnI-linearized pUD968 yielded plasmid pUDE885. Primers 16709 and 16710, with sequence homology to the 5′ and 3′ regions of the coding region of *SOR2* and to the 3′ and 5′ termini of the p*ACT1* and t*CPS1* sequences, respectively, were used to amplify the coding region of *SOR2* from genomic DNA of strain CEN.PK113-7D. Gibson assembly of KpnI-linearized pUDE885 with the resulting DNA fragment yielded pUDE941. Amplification of the p*ACT1*-*SOR2*-t*CPS1* expression cassette from pUDE941 with primer pair 16715/16716 added terminal sequences homologous to the X-2 integration site on Chromosome X of *S. cerevisiae* [52] and to synthetic homologous recombination sequence A (SHR-A). These terminal sequences allowed for simultaneous in vivo assembly and integration into the X-2 locus with the p*TEF1*-*HXT15*-t*CYC1* cassette.

### Yeast genome editing

The lithium acetate method [57] was used for yeast transformation. *S. cerevisiae* IMX2411 was constructed by transforming strain IMX581 with pUDR538, along with p*ACT1*-*SOR2*-t*CPS1*, p*TEF1*, *HXT15* and t*CYC1* DNA fragments. Transformants were selected on YPD plates (10 g L^−1^ Bacto yeast extract, 20 g L^−1^ Bacto peptone, 20 g L^−1^ glucose and 20 g L^−1^ agar) supplemented with 200 mg L^−1^ hygromycin B. After verification of correct assembly by diagnostic PCR, single colony isolates were restreaked thrice and stored. The empty-vector reference strain IME611 was constructed by transforming strain IMX2411 with p426-TEF (*URA3*). Uracil prototrophic transformants were selected on synthetic medium with vitamins [SM, [58]] supplemented with 20 g L^−1^ agar and 20 g L^−1^ sorbitol. A p*TEF1*-*HXT15*-t*CYC1* expression cassette was amplified from genomic DNA of strain IMX2411 with primer pair 16,711/16712. Strain IMX2495 was obtained by transforming strain IMX1489 with pUDR538 together with p*ACT1*-*SOR2*-t*CPS1* and p*TEF1*-*HXT15*-t*CYC1* fragments. Transformants were selected on YPD-hygromycin plates. Strain IMX2506 was obtained by transforming strain IMX2495 with p426-TEF (*URA3*). Transformants were selected on SM plates supplemented with 20 g L^−1^ sorbitol.

### Shake-flask cultivation

Aerobic shake-flask cultures were grown in 500-mL round-bottom shake flasks containing 100 mL medium, placed in an Innova incubator shaker (Eppendorf Nederland B.V., Nijmegen, The Netherlands) at 30 °C and 200 rpm. Anaerobic cultures were grown in 50-mL round-bottom shake flasks containing 30 mL medium, incubated at 30 °C in a Bactron anaerobic chamber (Sheldon Manufacturing Inc., Cornelius, OR) with an atmosphere of 5% (v/v) H_2_, 6% (v/v) CO_2_ and 89% (v/v) N_2_. Flasks were shaken on a IKA KS 260 basic shaker (Dijkstra Verenigde BV, Lelystad, The Netherlands) at 200 rpm [59].

### Bioreactor cultivation

Anaerobic chemostat cultures and batch cultures were grown at 30 °C in 2-L bioreactors (Applikon, Delft, The Netherlands), with 1-L working volume. Anaerobic chemostat cultures were grown at a dilution rate of 0.025 L h^−1^ and the effluent pump was controlled by an electrical level sensor to maintain a 1.0-L working volume. The pH was kept constant at 5.0 by automatic addition of 2 M KOH. Chemostat cultures were grown on SM with 10 g L^−1^ glucose and 10 g L^−1^ sorbitol and bioreactor batch cultures were grown on SM with 20 g L^−1^ glucose and 30 g L^−1^ sorbitol. Media was supplemented with the anaerobic growth factors Tween 80 (420 mg L^−1^) and ergosterol (10 mg L^−1^) [59], and 0.2 g L^−1^ antifoam C (Sigma-Aldrich). Unless otherwise indicated, cultures were sparged at 0.5 L min^−1^ with an N_2_/CO_2_ (90/10%) gas mixture. The outlet gas stream was cooled to 4 °C in a condenser to minimize evaporation. Oxygen diffusion was minimized by use of Norprene tubing (Saint-Gobain, Amsterdam, The Netherlands) and Viton O-rings (ERIKS, Haarlem, The Netherlands). Steady state was assumed when, after at least 5 volume changes of operation under constant conditions, biomass dry weight, ethanol, glycerol and acetate concentrations varied by less than 5% over at least two additional volume changes. Inocula for chemostat and bioreactor batch cultures were prepared in 500-mL shake flasks containing 100-mL SM with 20 g L^−1^ glucose. A first ‘wake-up’ culture was inoculated with frozen stock culture, grown aerobically for 15–18 h at 30 °C and used to inoculate precultures on SM. Upon reaching mid-exponential phase (OD_660_ of 3–5), these were used as inocula for bioreactor cultures at an initial OD_660_ of 0.2–0.3. Bioreactor batch cultures that preceded the chemostat-cultivation phase were grown on SM with 20 g L^−1^ glucose.

### Analytical methods

Biomass dry weight measurements, analysis of metabolite concentrations and correction for ethanol evaporation were performed as described previously [24]. Metabolite concentrations in steady-state chemostat cultures were analysed after rapid quenching of the culture samples using cold steel beads [60]. Carbon recoveries could not be accurately calculated due to the high concentration of CO_2_ in the inlet gas of bioreactor cultures of PRK-RuBisCO expressing strains [24, 26]. Instead, balances of degree of reduction of substrates and products [61] were calculated. These calculations were based on concentrations of relevant components in medium feed and culture samples and on a published value for the elemental composition of yeast biomass [62].

### High-throughput analysis of specific growth rates

A Growth-Profiler system (EnzyScreen, Heemstede, The Netherlands) was used for parallel analysis of specific growth rates of multiple *S. cerevisiae* strains. Cultures were grown under air on SM supplemented with either 20 g L^−1^ glucose or 20 g L^−1^ sorbitol, in a culture volume of 250 μL, at 30 °C and at an agitation rate of 250 rpm. The measurement interval was set at 30 min and specific growth rates were calculated from raw green values [63].

### Whole-genome sequencing

100 mL aerobic shake-flask cultures *S. cerevisiae* strains IMX1489 and IMX2506 on YPD were centrifuged for 10 min at 5,000 × *g* in late exponential phase (OD_660_ of 10–15). For genomic DNA isolation from chemostat cultures of strain IMX1489, 50 mL samples were harvested. Genomic DNA extracted with a Qiagen Blood & Cell Culture NDA kit and 100/G Genomics-tips (Qiagen, Hilden, Germany) was quantified with a Qubit Fluorometer 2.0 (Thermo Fisher). The genome of strain IMX1489 was sequenced in-house as described previously [64] on an Illumina Miseq sequencer (Illumina, San Diego, CA, USA) with a minimum of 50-fold read coverage. Custom paired-end sequencing of genomic DNA of duplicate chemostat cultures of IMX1489 was performed by Macrogen (Amsterdam, The Netherlands) on a 350-bp PCR-free insert library using Illumina SBS technology. Genomic DNA of strain IMX2506 was sequenced by Genomescan (Leiden, The Netherlands) with Illumina SBS technology yielding 151 bp reads with at least 50-fold read coverage. Sequence reads were mapped against the genome of *S. cerevisiae* CEN.PK113-7D [65] to which a virtual contig containing the sequences of pDAN-prk-tPGK1, pTDH3-cbbm-tCYC1, pTEF1-groEL-tACT1 and pTPI1-groES-tPGI1 had been added, and processed as described previously [64].

### Stoichiometric analysis

Quantitative estimates of the ethanol yield, biomass yield and biomass-specific rate of substrate consumption were obtained using a stoichiometric model of the core metabolic network of *S. cerevisiae* [29]. This model was adjusted as described previously, by including a PRK-RuBisCO-based bypass of the oxidative reaction in glycolysis (Fig. 1, [27]). For sorbitol-grown cultures, three additional reactions were implemented, corresponding to a sorbitol facilitator (Eq. 1), a sorbitol dehydrogenase (Eq. 2) and a fructose kinase (Eq. 3).

1 $$ {\text{sorbitol }}\left( {{\text{external}}} \right) \to {\text{sorbitol }}\left( {{\text{cytosolic}}} \right) $$

2 $$ {\text{sorbitol}} + {\text{NAD}} + \to {\text{fructose}} + {\text{NADH}} $$

3 $$ {\text{fructose}} + {\text{ATP}} \to {\text{fructose}} - {\text{6P}} + {\text{ADP}} $$

## Supplementary Information

**Additional file 1.** Measurement data used to prepare Fig. 3.

**Additional file 2.** Measurement data used to prepare Tables 1 and 2.

**Additional file 3.** Measurement data used to prepare** Figure S2**.

**Additional file 4.** Measurement data used to prepare** Tables S2** and** S3**.

**Additional file 5: Table S1. **Predicted ethanol yields on substrate, biomass-specific substrate-uptake rates (q_substrate_) and biomass yields on substrate for wild-type *S. cerevisiae *(WT) and strains with an engineered PRK-RuBisCO bypass of the oxidative reaction in glycolysis on both glucose and on sorbitol. Rates and yields were predicted for cultures growing at different specific growth rates, using an extended stoichiometric model of the core metabolic network of *S.* *cerevisiae* (1, 2). A Cmol biomass (CH_1.8_O_0.5_N_0.2_, (3)) corresponds to 26.4 g dry biomass. **Table S2** Maximum specific growth rates in aerobic batch cultures of *S.* *cerevisiae strains* IMX2506 (*gpd2∆* {PRK-RuBisCO} *HXT15*↑ *SOR2*↑) and IME611 (*GPD2* *HXT15*↑ *SOR2*↑) on synthetic medium, supplemented with either 20 g L^-1^ of glucose or 20 g L^-1^ of sorbitol. Specific growth rates were calculated from quadruplicate cultures in a Growth Profiler, using at least 9 measurement points obtained during the exponential growth phase. Strain IME324 (*GPD2*) was inoculated in duplicate aerobic shake-flask cultures containing synthetic medium supplemented with 20 g L^-1^ of sorbitol as sole carbon source. No growth was observed after 4 weeks of incubation. N.D.: not determined. **Table S3 **Segmental aneuploidies observed in two prolonged anaerobic chemostat cultivation experiments with *S. cerevisiae* IMX2506 (*gpd2∆ *{PRK-RuBisCO} *HXT15*↑ *SOR2*↑) on glucose- sorbitol mixtures (see Fig. 5). **Table S4** Oligonucleotide primers used in this study. **Figure S1A** Copy number variation across yeast chromosomes in prolonged anaerobic chemostat cultivation experiment 1 with *S. cerevisiae* IMX2506 (*gpd2∆ *{PRK-RuBisCO} *HXT15*↑ *SOR2*↑) on glucose- sorbitol mixtures (see Fig. 5). Copy number variations were visualized with the Magnolya algorithm (4). **Figure S1B** Copy number variation across yeast chromosomes in prolonged anaerobic chemostat cultivation experiment 2 with *S. cerevisiae* IMX2506 (*gpd2∆ *{PRK-RuBisCO} *HXT15*↑ *SOR2*↑) on glucose–sorbitol mixtures (see Fig. 5). Copy number variations were visualized with the Magnolya algorithm (4). **Figure S1C **Reference data for copy-number assessment with the Magnolya algorithm (4) for the reference strain *S. cerevisiae* IMX2506 (*gpd2∆* {PRK-RuBisCO} *HXT15*↑ *SOR2*↑). **Figure S2** Optical density at 660 nm (OD_660_), sorbitol concentration, ethanol concentration and glycerol concentration in duplicate aerobic shake-flask cultures of S. cerevisiae IMX2506 (gpd2∆ {PRK-RuBisCO} HXT15↑ SOR2↑) on 20 g L^-1^ sorbitol.

## Data Availability

DNA sequencing data of the *Saccharomyces cerevisiae* strains IMX1489, IMX2506 and evolved population of evolution line 1 (deposited as IMS1232) and evolution line 2 (deposited as IMS1233) were deposited at NCBI under BioProject accession number PRJNA818459. All measurement data used to prepare Fig. 3, Tables 1 and 2, Fig. 5, Figure S2 and Tables S2 and S3 of the manuscript are available in Additional file 1, Additional file 2, Additional file 3 and Additional file 4, respectively.
