# Supplementary material for: An engineered non-oxidative glycolytic bypass based on Calvin-cycle enzymes enables anaerobic co-fermentation of glucose and sorbitol by Saccharomyces cerevisiae
Source: Biotechnol Biofuels Bioprod. 2022 Oct 17;15:112. doi: 10.1186/s13068-022-02200-3 (PMC9578259; doi:10.1186/s13068-022-02200-3)
Supplement: Supplementary file 5 — Additional file 5: Table S1. Predicted ethanol yields on substrate, biomass-specific substrate-uptake rates (qsubstrate) and biomass yields on substrate for wild-type S. cerevisiae (WT) and strains with an engineered PRK-RuBisCO bypass of the oxidative reaction in glycolysis on both glucose and on sorbitol. Rates and yields were predicted for cultures growing at different specific growth rates, using an extended stoichiometric model of the core metabolic network of S. cerevisiae (1, 2). A Cmol biomass (CH1.8O0.5N0.2, (3)) corresponds to 26.4 g dry biomass. Table S2 Maximum specific growth rates in aerobic batch cultures of S. cerevisiae strains IMX2506 (gpd2∆ {PRK-RuBisCO} HXT15↑ SOR2↑) and IME611 (GPD2 HXT15↑ SOR2↑) on synthetic medium, supplemented with either 20 g L-1 of glucose or 20 g L-1 of sorbitol. Specific growth rates were calculated from quadruplicate cultures in a Growth Profiler, using at least 9 measurement points obtained during the exponential growth phase. Strain IME324 (GPD2) was inoculated in duplicate aerobic shake-flask cultures containing synthetic medium supplemented with 20 g L-1 of sorbitol as sole carbon source. No growth was observed after 4 weeks of incubation. N.D.: not determined. Table S3 Segmental aneuploidies observed in two prolonged anaerobic chemostat cultivation experiments with S. cerevisiae IMX2506 (gpd2∆ {PRK-RuBisCO} HXT15↑ SOR2↑) on glucose- sorbitol mixtures (see Fig. 5). Table S4 Oligonucleotide primers used in this study. Figure S1A Copy number variation across yeast chromosomes in prolonged anaerobic chemostat cultivation experiment 1 with S. cerevisiae IMX2506 (gpd2∆ {PRK-RuBisCO} HXT15↑ SOR2↑) on glucose- sorbitol mixtures (see Fig. 5). Copy number variations were visualized with the Magnolya algorithm (4). Figure S1B Copy number variation across yeast chromosomes in prolonged anaerobic chemostat cultivation experiment 2 with S. cerevisiae IMX2506 (gpd2∆ {PRK-RuBisCO} HXT15↑ SOR2↑) on glucose–sorbitol mixtures (see Fig [file 13068_2022_2200_MOESM5_ESM.docx]

**Supplementary Materials**

**An engineered non-oxidative glycolytic bypass based on Calvin-cycle enzymes enables anaerobic co-fermentation of glucose and sorbitol by *Saccharomyces cerevisiae***

Aafke C.A. van Aalst, Robert Mans and Jack T. Pronk

**Table S1** Predicted ethanol yields on substrate, biomass-specific substrate-uptake rates (q_substrate_) and biomass yields on substrate for wild-type *S. cerevisiae* (WT) and strains with an engineered PRK-RuBisCO bypass of the oxidative reaction in glycolysis on both glucose and on sorbitol. Rates and yields were predicted for cultures growing at different specific growth rates, using an extended stoichiometric model of the core metabolic network of *S. cerevisiae* (1, 2). A Cmol biomass (CH_1.8_O_0.5_N_0.2_, (3)) corresponds to 26.4 g dry biomass.

| Specific growth rate (h^-1^) | **Y_ethanol/substrate_ (mol/mol)** | | | **q_substrate_ (mmol/Cmol/h)** | | | **Y_x/substrate_ (mol/mol)** | | |
| --- | --- | --- | --- | --- | --- | --- | --- | --- | --- |
|  | WT | PRK-RuBisCO | | WT | PRK-RuBisCO | | WT | PRK-RuBisCO | |
|  | glucose | glucose | sorbitol | glucose | glucose | sorbitol | glucose | glucose | sorbitol |
| 0.0001 | 2.00 | 2.00 | 2.17 | 13.3 | 13.3 | 22.8 | 0.008 | 0.008 | 0.004 |
| 0.001 | 1.95 | 1.98 | 2.15 | 14.5 | 14.2 | 24.3 | 0.069 | 0.071 | 0.041 |
| 0.01 | 1.75 | 1.86 | 2.09 | 26.2 | 23.0 | 39.5 | 0.382 | 0.434 | 0.253 |
| 0.03 | 1.62 | 1.78 | 2.04 | 52.1 | 42.7 | 73.0 | 0.576 | 0.703 | 0.410 |
| 0.1 | 1.54 | 1.71 | 2.00 | 143 | 111 | 191 | 0.701 | 0.898 | 0.524 |
| 0.2 | 1.52 | 1.70 | 1.99 | 272 | 210 | 359 | 0.735 | 0.954 | 0.557 |
| 0.3 | 1.51 | 1.69 | 1.99 | 402 | 308 | 528 | 0.747 | 0.975 | 0.569 |

**Table S2**  Maximum specific growth rates in aerobic batch cultures of *S. cerevisiae strains* IMX2506 (*gpd2∆* {PRK-RuBisCO} *HXT15↑ SOR2↑*) and IME611 (*GPD2* *HXT15*↑ *SOR2*↑) on synthetic medium, supplemented with either 20 g L^-1^ of glucose or 20 g L^-1^ of sorbitol. Specific growth rates were calculated from quadruplicate cultures in a Growth Profiler, using at least 9 measurement points obtained during the exponential growth phase. Strain IME324 (*GPD2*) was inoculated in duplicate aerobic shake-flask cultures containing synthetic medium supplemented with 20 g L^-1^ of sorbitol as sole carbon source. No growth was observed after 4 weeks of incubation. N.D.: not determined.

| **strain** | **Relevant genotype** | **Specific growth rate on glucose (h^-1^)** | **Specific growth rate on sorbitol (h^-1^)** |
| --- | --- | --- | --- |
| **IME324** | *GPD2* | N.D. | <0.001 |
| **IME611** | *GPD2* *HXT15*↑ *SOR2*↑ | 0.36 ± 0.01 | 0.23 ± 0.00 |
| **IMX2506** | *gpd2*∆ non-ox PPP↑ p*DAN1*-*prk* *cbbm* *HXT15*↑ *SOR2*↑ | 0.35 ± 0.01 | 0.25 ± 0.00 |

**Table S3** Segmental aneuploidies observed in two prolonged anaerobic chemostat cultivation experiments with *S. cerevisiae* IMX2506 (*gpd2∆* {PRK-RuBisCO} *HXT15*↑ *SOR2*↑) on glucose- sorbitol mixtures (see Fig. 5).

| **Partial aneuploidy on chromosome and coordinates** | **Found in** | **Genes located on duplicated region** |
| --- | --- | --- |
| IV (~1,075,000 – 1,185,000) | Evolution experiment 1  Evolution experiment 2 | *OMS1*, *HIM1*, *MCM21*, *YFT2*, *SWA2*, *DAD4*, *ASP1*, *MRPL35*, *TIM11*, *PEP7*, *UTP4*, *YCG1*, *YSP*2, *SKP1*, *PEX3*, *UBX5*, *GPI8*, *IRC3*, *YDR333C*, *SWR1*, *MSN5*, *YDR336W*, *MRPS28*, *YDR338C*, *FCF1*, *YDR341C*, *HXT7*, *HXT6*, *HXT3*, *SVF1*, *MRP1*, *PAL1*, *YPS7*, *ATP22*, *SBE2*, *YDR352W*, *TRR1*, *TRP4*, *SPC110*, *CLN1*, *GGA1*, *EAF1*, *BCP1* |
| VIII (~250,000-end) | Evolution experiment 2 | >100 genes |
| IX (~180,000-215,000) | Evolution experiment 2 | *PRK1*, *LYS12*, *RSM25*, *YIL092W*, *UTP25*, *ICE2*, *YIL089W*, *AVT7*, *AIM19*, *KTR7*, *SDS3*, *THS1*, *YIL077C*, *SEC28*, *RPN2*, *SER33*, *SPO22* |
| X (~415,00-435,00) | Evolution experiment 1 | *CYR1*, *SYS1*, *COX16*, *OST1*, *PRE3*, *AVT1*, *MPP10*, *YJR003C*, *SAG1* |
| XV (~0-200,000) | Evolution experiment 1  Evolution experiment 2 | >100 genes |
| XV (~215,000-495,000) | Evolution experiment 1  Evolution experiment 2 | >100 genes |


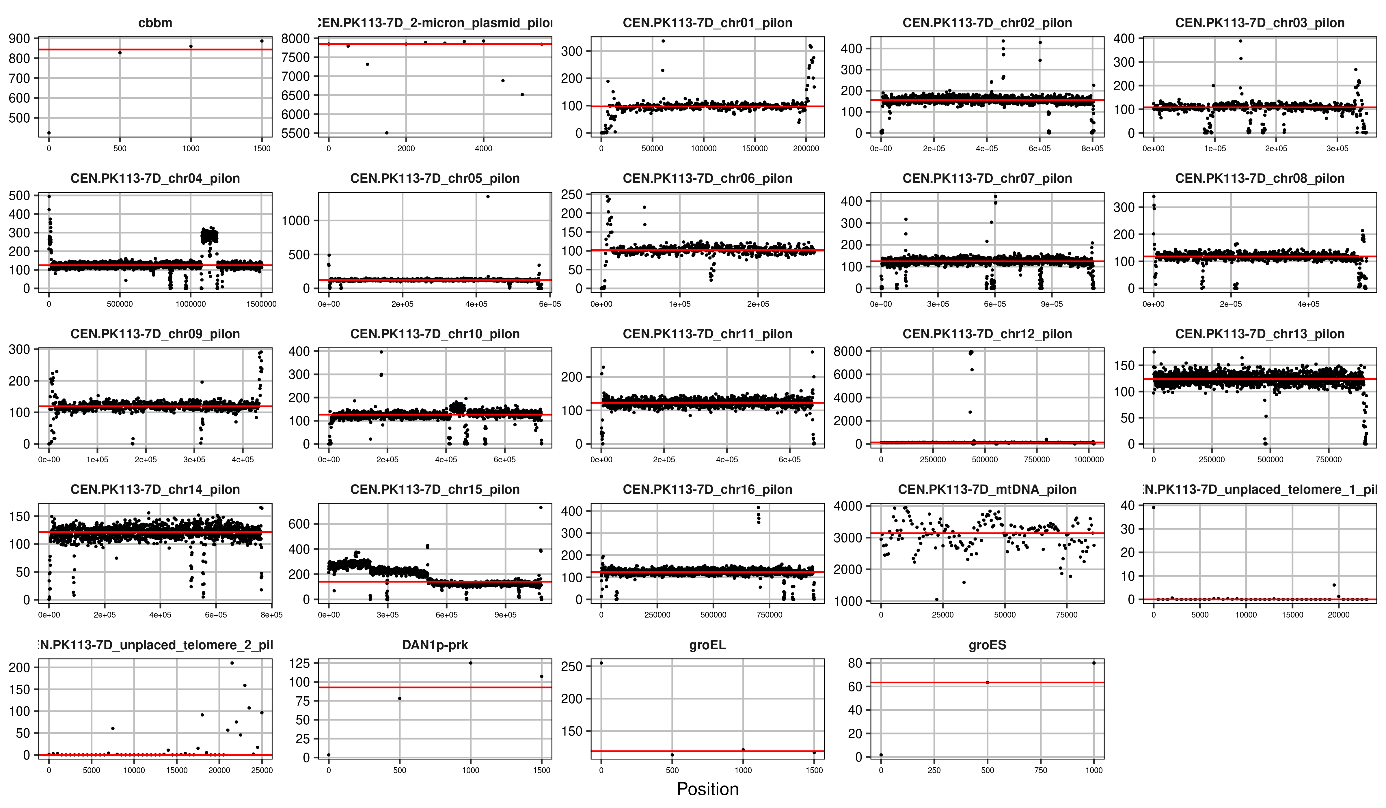


**Figure S1A** Copy number variation across yeast chromosomes in prolonged anaerobic chemostat cultivation experiment 1 with *S. cerevisiae* IMX2506 (*gpd2∆* {PRK-RuBisCO} *HXT15*↑ *SOR2*↑) on glucose- sorbitol mixtures (see Fig. 5). Copy number variations were visualized with the Magnolya algorithm (4).


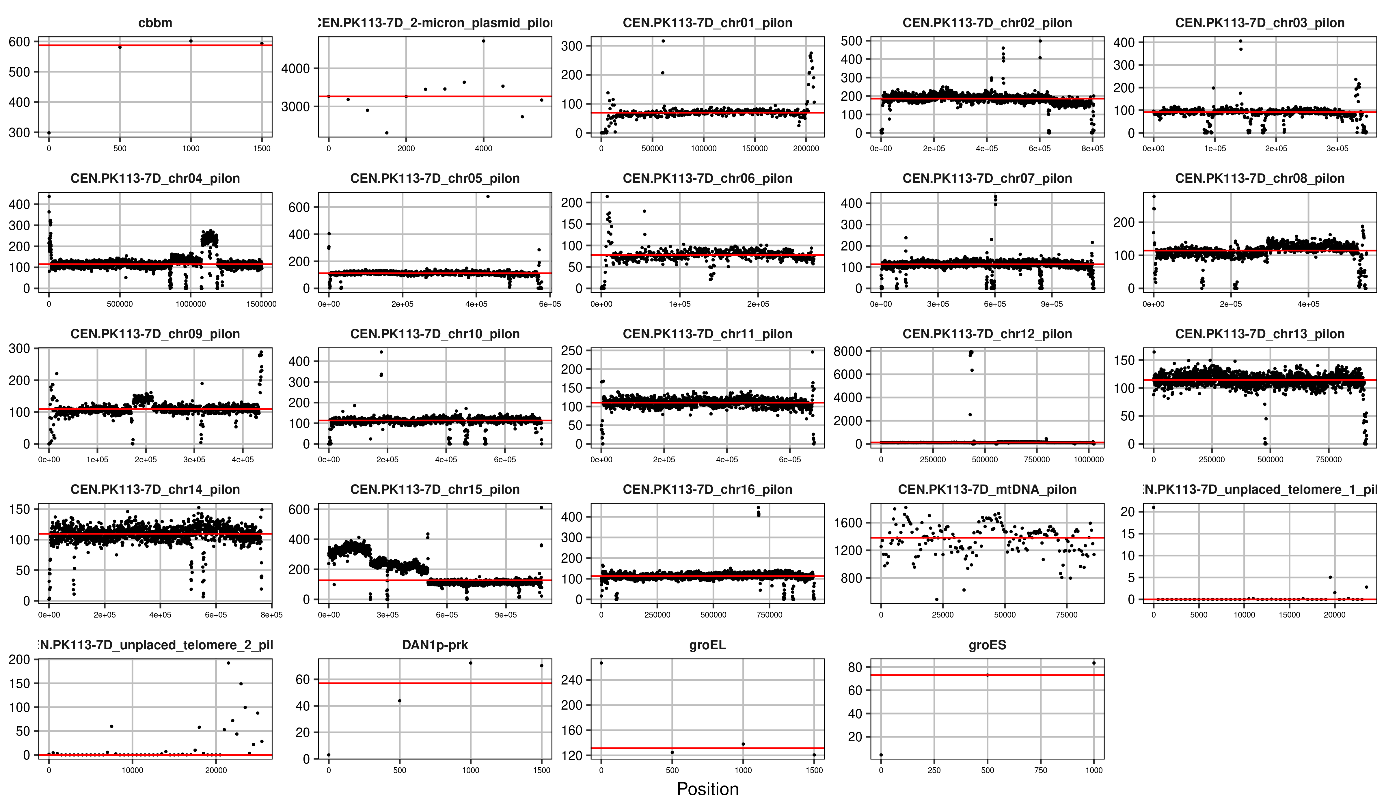


**Figure S1B** Copy number variation across yeast chromosomes in prolonged anaerobic chemostat cultivation experiment 2 with *S. cerevisiae* IMX2506 (*gpd2∆* {PRK-RuBisCO} *HXT15*↑ *SOR2*↑) on glucose- sorbitol mixtures (see Fig. 5). Copy number variations were visualized with the Magnolya algorithm (4).


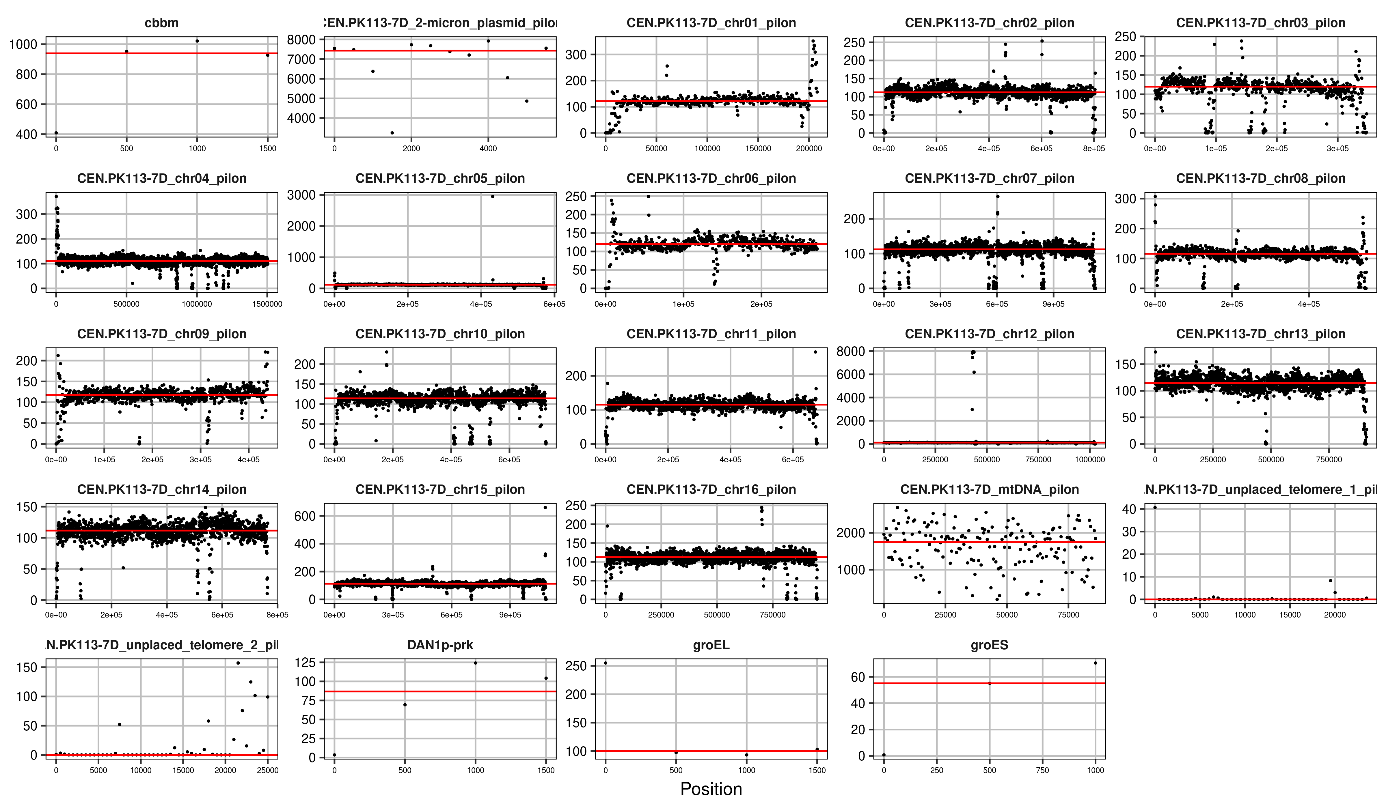


**Figure S1C** Reference data for copy-number assessment with the Magnolya algorithm (4) for the reference strain *S. cerevisiae* IMX2506 (*gpd2∆* {PRK-RuBisCO} *HXT15*↑ *SOR2*↑).


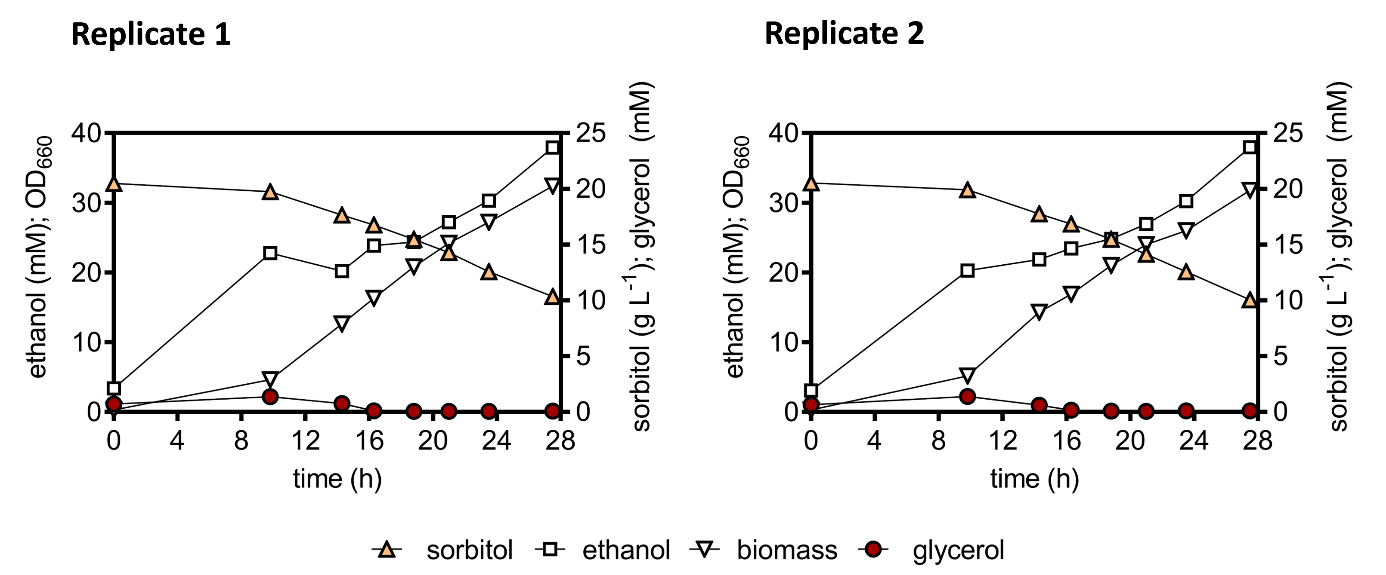


**Figure S2** Optical density at 660 nm (OD_660_), sorbitol concentration, ethanol concentration and glycerol concentration in duplicate aerobic shake-flask cultures of S. cerevisiae IMX2506 (gpd2∆ {PRK-RuBisCO} HXT15↑ SOR2↑) on 20 g L^-1^ sorbitol.

**Table S4** Oligonucleotide primers used in this study.

| Primer | Description | Sequence | Purification |
| --- | --- | --- | --- |
| 7376 | X-2 outside fw | GGTCTAGGCCTGCATAATCG | DST |
| 7377 | X-2 outside rev | TGCGGCATCATGTCTACTTG | DST |
| 7388 | p426 backbone | AGGCGTATCACGAGGCCCTTTC | PAGE |
| 10901 | p426 backbone | AAGTCTGTGCTCCTTCCTTC | DST |
| 15514 | p426 backbone | AAAGGTACCCGGTCAGATGGGATACAATCTAGATAAGTTGCGCTGTAGCAGCAAGCTGAATAGCGATGCGCATTCAGCTCCAGCTTTTGTTCCCTTTAG | DST |
| 15515 | p426 backbone | AAAGGTACCATAGCTTCCGGCACATGGTGAGATGCACTCTAACCGTCAGGCGACGTATAGATTCGTATGGCATTCTACCCAATTCGCCCTATAGTGAG | DST |
| 15548 | pACT1 fw | GATTGTATCCCATCTGACCGCCCTAACATATTTTCCAATTAACCCTCAATATTTC | DST |
| 15549 | pACT1 rev | GGTACCCATCTGAATAGGTACCACCAGAACCGTTATCAATAACCAAAGC | DST |
| 15550 | tCPS1 rev | CACCATGTGCCGGAAGCTATTTATCATCATCATTTAAATTTTGATTTGACACTTGATTTG | DST |
| 15551 | tCPS1 fw | GGTACCTATTCAGATGGGTACCGCGCAATGATTGAATAGTCAAAG | DST |
| 16705 | pTEF1_HXT15 fw | TACAACTTTTTTTACTTCTTGCTCATTAGAAAGAAAGCATAGCAATCTAATCTAAGTTTTATGGCAAGCGAACAGTCCTCACCAGAAATT | DST |
| 16706 | tCYC1_HXT15 rev | CGGTTAGAGCGGATGTGGGGGGAGGGCGTGAATGTAAGCGTGACATAACTAATTACATGATCAATTAAAACTCTTTGGGAACTTCAAAAC | DST |
| 16709 | pACT1_SOR2 fw | TTTTGTTGCTATATTATATGTTTAGAGGTTGCTGCTTTGGTTATTGATAACGGTTCTGGTATGTCTCAAAATAGTAACCCTGCAGTAGTT | DST |
| 16710 | tCPS1_SOR2 rev | GTTCTATGAAAAAAAAAAAAAATTAAAAAAAAAAAATCTTTGACTATTCAATCATTGCGCTCATTCAGGACCAAAGATAATAGTCTTGAC | DST |
| 16711 | X-2_pTEF1 fw | TCACAGAGGGATCCCGTTACCCATCTATGCTGAAGATTTATCATACTATTCCTCCGCTCGCATAGCTTCAAAATGTTTCTACTCCTTTTT | DST |
| 16712 | SHR-A_tCYC1 rev | CCGTGCCATAGCCATGCCTTCACATATAGTGCCGCAAATTAAAGCCTTCGAGCGTCCCAAAACCTTCTCA | DST |
| 16715 | SHR-A_pACT1 fw | ACTATATGTGAAGGCATGGCTATGGCACGGCCCTAACATATTTTCCAATTAACCCTCAATATTTCTCTGT | DST |
| 16716 | X-2_tCPS1 rev | GTCATAACTCAATTTGCCTATTTCTTACGGCTTCTCATAAAACGTCCCACACTATTCAGGGGTTTATCATCATCATTTAAATTTTGATTTGAC | DST |
| 17031 | HXT15_pTEF1 rev | AGCTGCACTACTGTTTAGATTATCTGCATTAATTTCTGGTGAGGACTGTTCGCTTGCCATAAAACTTAGATTAGATTGCTATGCTTTCTT | DST |
| 17032 | HXT15_tCYC1 fw | TTTGAAGTTCCCAAAGAGTTTTAATTGATCATGTAATTAGTTATGTCACGCTTACATTCACGCCCTCCCCCCACATCCGCTCTAACCGAA | DST |

**References**

1. van Aalst AC, de Valk SC, van Gulik WM, Jansen ML, Pronk JT, Mans R. Pathway engineering strategies for improved product yield in yeast-based industrial ethanol production. Synthetic and Systems Biotechnology. 2022;7:554-66.

2. Daran-Lapujade P, Jansen ML, Daran J-M, van Gulik W, de Winde JH, Pronk JT. Role of transcriptional regulation in controlling fluxes in central carbon metabolism of *Saccharomyces cerevisiae*: a chemostat culture study. Journal of Biological Chemistry. 2004;279:9125-38.

3. Lange H, Heijnen J. Statistical reconciliation of the elemental and molecular biomass composition of *Saccharomyces cerevisiae*. Biotechnology and bioengineering. 2001;75:334-44.

4. Nijkamp JF, van den Broek M, Datema E, de Kok S, Bosman L, Luttik MA, et al. De novo sequencing, assembly and analysis of the genome of the laboratory strain *Saccharomyces cerevisiae* CEN. PK113-7D, a model for modern industrial biotechnology. Microbial cell factories. 2012;11:1-17.
